# Enhanced liver fibrosis score as a noninvasive biomarker in hepatitis C virus patients after direct-acting antiviral agents

**DOI:** 10.3389/fphar.2022.891398

**Published:** 2022-08-17

**Authors:** Valentina Cossiga, Evelina La Civita, Dario Bruzzese, Maria Guarino, Andrea Fiorentino, Rosanna Sorrentino, Giuseppina Pontillo, Luca Vallefuoco, Stefano Brusa, Emma Montella, Daniela Terracciano, Filomena Morisco, Giuseppe Portella

**Affiliations:** ^1^ Department of Clinical Medicine and Surgery, University of Naples “Federico II”, Naples, Italy; ^2^ Department of Translational Medical Science, University of Naples “Federico II”, Naples, Italy; ^3^ Department of Public Health, University of Naples Federico II, Naples, Italy

**Keywords:** ELF index, HCV, liver fibrosis, transient elastography (FibroScan), direct-acting agents

## Abstract

**Background:** In more than 90% of chronic viral hepatitis C (HCV) patients treated with direct-acting antiviral agents (DAAs), a sustained viral response (SVR) was observed. Unfortunately, there are subgroups of subjects who display enduring liver fibrosis and are at high risk of developing hepatocellular carcinoma (HCC). Thus, liver fibrosis evaluation during the follow-up of these patients plays a pivotal role. The gold standard to evaluate hepatic fibrosis is liver biopsy, which is an invasive procedure. Imaging techniques and serum biomarkers have been proposed as safer and cheaper procedures.

**Objectives:** In this study, we evaluated the concordance of transient elastography (TE) with ELF score ( enhanced liver fibrosis) in a cohort of patients with HCV before and after direct-acting antiviral (DAAs) treatment. ELF score has been validated in other chronic liver diseases; the evidence is not available in HCV patients treated with DAAs.

**Study design:** We prospectively recruited all consecutive HCV patient candidates for DAAs therapy at the University of Naples “Federico II” between April 2015 and July 2016. TE and ELF scores were assessed at baseline, at SVR24, and at SVR48.

**Results:** One-hundred-nineteen patients were treated with DAAs, and 94.1% of them reached SVR. A total of 55.5% of patients were males with a mean age of 64.7 ± 9.6 years. TE results revealed that 12 patients (10%) had F1-2 mild/moderate fibrosis, and 107 (90%) had F3-4 advanced fibrosis. At baseline, SVR24, and SVR48, the concordance between ELF test and TE was poor: 0.11 (*p* = 0.086), 0.15 (*p* = 0.124), and 0.034 (*p* = 0.002), respectively. However, at SVR24 and SVR48, both methods showed a significant amelioration of liver fibrosis compared to baseline (*p* < 0.001). In addition, both ELF index and TE were significantly associated with portal hypertension at baseline, but not with varices and ascites.

**Conclusions:** Our findings suggested that ELF test could predict changes in liver fibrosis, independently of TE. In case of TE unavailability, ELF score could represent an appropriate tool. Notably, in the context of the COVID-19 pandemic, ELF testing should be encouraged to reduce unnecessary access to the hospital and prolonged physical contact.

## Introduction

Direct-acting antiviral agents (DAAs) represent a milestone in the clinical management of chronic hepatitis C virus (HCV) patients. More than 90% of these subjects reach sustained virologic response (SVR) and effectively recover (Kowdley et al., 2014; Lawitz et al., 2014; Muir et al., 2015). However, in some patients, liver inflammation and fibrosis persist after DAA treatment (Putra et al., 2018), and SVR might not correlate with a reduced risk of hepatocellular carcinoma (HCC) (Sapena et al., 2022). Therefore, assessment of liver fibrosis in chronic hepatitis C virus (HCV) infection is crucial to monitor the response to treatment, the progression of liver damage, and to program an adequate follow-up (Carmona et al., 2016).

The current gold standard to evaluate hepatic fibrosis is liver biopsy, an invasive procedure, poorly tolerated by patients and carrying a small but significant risk of complications. Moreover, the specimen of liver biopsy can be limited by sampling error, and with a significant intra- and inter-observer variability in the assessment of fibrosis stages (Regev et al., 2002; Rockey et al., 2009). In a study, up to 10% variability in the staging of the same specimen after repeated assessments by a single observer was reported (Vuppalanchi et al., 2009). Not surprisingly, noninvasive tests (NTIs) have been identified and validated to indirectly estimate liver fibrosis (Krag et al., 2022). NTIs to identify fibrosis stage allow performing serial follow-up of patients or, in the case of viral hepatitis, to assess therapy response (Stasi and Milani, 2016; Wong, 2018). NTIs comprise imaging techniques and circulating biomarkers (Sharma et al., 2014a). Among imaging approaches, transient elastography (FibroScan^®^) is one of the most commonly used, showing a high correlation to liver biopsy for staging hepatic fibrosis (Arena et al., 2008) and providing accurate diagnostic and prognostic information (Castera, 2011; Fernandez et al., 2015). Unfortunately, its availability is scarce and requires trained personnel (Fraquelli et al., 2007; Castera et al., 2010). This technique has several limitations, including the cost of the equipment and the lack of standardized cut-offs for the diagnosis of fibrosis stages. Despite these limitations, TE is currently the second-best option for staging hepatic fibrosis.

Serum biomarkers and biomarker scores have also been proposed for the assessment of fibrosis. Biomarker scores are highly reproducible, cost-effective, and do not require trained personnel or expensive instruments (Wong, 2018).

The enhanced liver fibrosis (ELF) score is one of the most widely studied (Patel et al., 2020; Abdel-Hameed et al., 2021). The algorithm of ELF score combines three serum markers of fibrosis: hyaluronic acid (HA); amino-terminal propeptide of type-III-procollagen (PIIINP); and tissue inhibitor of metalloproteinase type-1 (TIMP-1).

Elevated HA levels reflect increased production of HA within a fibrotic liver or a reduced clearance. Elevated TIMP-1 levels were observed in alcoholic patients with fibrosis and cirrhosis. PIIINP (amino-terminal of serum procollagen Ⅲ peptide) is a marker of collagen turnover. Increased levels occur in tissue repair and fibrosis (Sharma et al., 2014b).

Overall, these biomarkers are involved directly in the synthesis and degradation of hepatic extracellular matrix (Patel et al., 2020); thus, it can be assumed that ELF score more directly mirror the extracellular matrix (ECM) turnover, the central event of hepatic fibrosis.

So far, ELF score has been validated in patients with chronic liver diseases, non-alcoholic steatohepatitis (NASH), hepatitis B, and hepatitis C (Guha et al., 2008; Parkes et al., 2010; Parkes et al., 2011; Trembling et al., 2014; Fernandes et al., 2015; Thiele et al., 2018).

No evidence is available on ELF score in subjects with HCV treated with DAAs. In this study, we evaluated ELF score to assess the modification of liver fibrosis in patients with HCV chronic hepatitis before and after DAAs treatment. TE was used as a reference assay to assess liver fibrosis, as most patients were not compliant with liver biopsy.

## Patients and methods

### Study design and patient population

From April 2015 to July 2016, 119 patients with a diagnosis of chronic hepatitis HCV treated with IFN-free DAA (direct-acting antiviral) regimens were prospectively and consecutively enrolled at the Liver Unit of the University of Naples “Federico II.” The study was conducted in accordance with the Declaration of Helsinki. The protocol was approved by the local ethics board of the promoting center (Federico II University of Naples, n 245/2013). All patients and controls involved in the study provided written informed consent to participate. Exclusion criteria included patients with current or past HCC and history of other malignancies, HBV or HIV co-infection, liver transplant recipients, and pregnancy or breastfeeding.

Demographic and laboratory data, comorbidities, and information regarding liver disease were collected. All patients were treated with DAA regimens available according to AISF (Italian Association for the Study of Liver) and EASL (European Association for the Study of the Liver) guidelines (European, 20182018). DAA regimens employed for the treatment of HCV infection are shown in Supplementary Table S1.

Liver fibrosis was evaluated with both TE and ELF tests at baseline, 24 weeks after DAA treatment (SVR24), and 48 weeks after DAA treatment (SVR48). Enrolled patients submitted to TE by Fibroscan^®^ and to a fasting blood sample on the same days. Patients were divided into two groups, according to TE: the F1-F2 group with mild/moderate fibrosis and the F3-F4 group with advanced fibrosis.

Portal hypertension was assessed considering the direct presence of gastroesophageal varices, portal hypertensive gastropathy, and/or indirect (liver stiffness >25 kPa, splenomegaly, and thrombocytopenia) signs of clinically significant portal hypertension (CSPH).

The study protocol was approved by the Ethics Committee of the University of Naples “Federico II.” All the study’s procedures were conducted according to the provisions of the Declaration of Helsinki and Good Clinical Practice Guidelines.

### Transient elastography measurements

Liver stiffness measurements (LSM) were performed by a single well-trained operator using a TE-FibroScan instrument (502Touch, EchosenseTM, Paris, France). The results were expressed in kiloPascals (kPa) with a range from 2.5 to 75 kPa. IQR was defined as an index of the intrinsic variability of LSM. Only those measurements with more than ten successful acquisitions, with a success rate of at least 60% and an interquartile range lower than 30%, were classified as valid and taken into consideration for statistical evaluation (Nitta et al., 2009).

In HCV patients, LSM correlates strongly with METAVIR fibrosis stages (de Ledinghen and Vergniol, 2008). In this study, the F1-F2 group was defined by LSM <10 kPa, while the F3-F4 group was identified by LSM >10 kPa.

### Enhanced liver fibrosis test

Fasting blood samples were obtained. All sera were frozen and stored at −20°C until measurement. Samples were assayed in an automated analyzer that performs magnetic separation enzyme immunoassay tests (ADVIA Centaur; Siemens Healthcare Diagnostics, Tarrytown, NY). Results were entered into the manufacturer’s published algorithm to derive an ELF score [ELF = 2.278 + 0.851 ln (HA) + 0.751 ln (PIIINP) +0.394 ln (TIMP-1)].

The cutoff points suggested by the manufacturer were <7.7, none to mild fibrosis; 7.7 to <9.8, moderate fibrosis; and >9.8, severe fibrosis (Day et al., 2019). In our study, the F1-F2 group was defined by ELF score values <9.8 and the F3-F4 group by values >9.8.

### Statistical analysis

All statistical analyses were performed using R Language for Statistical Computing (version 4.0.3). Continuous variables were expressed as mean ± standard deviation (SD) with range or, in the case of skewed variables, as median (25^th^ and 75th percentile) with range; qualitative variables were reported as absolute frequency and percentage. Accordingly, between-group comparisons were based either on the t-test for independent samples and the Mann–Whitney U-test or the chi-square test and the Fisher exact test (when appropriate). The concordance between TE and ELF scores was assessed both by Spearman correlation coefficient and by Cohen's kappa with the corresponding 95% confidence interval (95% CI). Assessment of time trends in the severity of fibrosis was based on the McNemar test for paired samples. All tests were two-sided with *p*-value < 0.05 denoting statistical significance.

## Results

### General characteristics of the study population

One-hundred-nineteen patients were enrolled and treated with DAAs from April 2015 to July 2016 at the Liver Unit of University Hospital of Naples Federico II. Most of them (94.1%) showed sustained virological response (SVR). Baseline demographic, clinical, and laboratory characteristics of the patients, stratified in the F1-F2 and F3-F4 groups according to TE values, are summarized in Table 1. According to liver fibrosis, at baseline, 12/119 (10.1%) patients were in the F1-F2 group and 107/119 (89.9%) in the F3-F4 group. The different distribution was due to HCV treatment criteria effective at the time of the enrollment. A total of 55.5% of patients were males with a mean age of 64.7 ± 9.6 (range: 31.1–81.9) years at the start of therapy. The mean BMI was 26.5 ± 3.6 kg/m^2^. The age and BMI were significantly higher in the F3-F4 group (*p* = 0.031 and *p* = 0.032, respectively) than those in the F1-F2 group. Seventy (58.8%) patients were interferon experienced without a significant difference between the two groups. As expected, at baseline, in the F3-F4 group, alfa-fetoprotein (AFP) levels were higher than in the F1-F2 group (*p* = 0.003), while albumin levels were significantly lower (*p* = 0.036).

**TABLE 1:** Baseline demographic, clinical, and laboratory characteristics of 119 patients; overall and stratified to liver fibrosis according to TE values.

	Overall	F1-F2	F3-F4	*p*-value
	(*n* = 119)	(*n* = 12)	(*n* = 107)	
Gender, male	66 (55.5)	6 (50)	60 (56.1)	0.924
Age, years	64.7 ± 9.6 (31.1–81.9)	55.9 ± 13.5 (31.1–73.9)	65.7 ± 8.6 (32.7–81.9)	0.031
BMI, kg/m^2^	26.5 ± 3.6 (18.3–37.9)	24.6 ± 2.8 (18.3–28.9)	26.7 ± 3.6 (18.3–37.9)	0.032
Previously treated	70 (58.8)	6 (50)	64 (59.8)	0.548
Albumin, g/dl	3.9 ± 0.4 (2.8–5)	4.2 ± 0.5 (3.4–5)	3.8 ± 0.4 (2.8–4.7)	0.036
INR	1.1 ± 0.2 (0.9–2.7)	1.1 ± 0.1 (0.9–1.4)	1.1 ± 0.2 (0.9–2.7)	0.209
Bilirubin, mg/dl	0.9 ± 0.5 (0.2–2.6)	0.7 ± 0.4 (0.2–1.7)	1 ± 0.5 (0.3–2.6)	0.098
Platelets as 10^3^/L	130 (40–549)	152 (47–321)	129 (40–549)	0.171
AST, IU/L	68 (18–288)	38 (18–77)	73 (23–288)	0.001
ALT, IU/L	71 (19–412)	45 (26–112)	73 (19–412)	0.057
AFP, ng/ml	9.2 (0.1–156.9)	3.8 (0.1–13)	10.2 (1.9–156.9)	0.003

Data represent mean ± standard deviation (range); median (IQR) (range) or n (%).

INR, international normalized ratio; AST, aspartate-aminotransferase; ALT, alanine-aminotransferase; AFP, alfa-fetoprotein; IQR, interquartile range.

### Enhanced liver fibrosis and transient elastography concordance

One-hundred-four patients were considered for the analysis, and 15 patients were excluded as both tests were not available.

When considering ELF and TE measures in their original numerical scale, a weak, although significant, correlation was observed in all time points (baseline: r = 0.335, *p* < 0.001; SVR24: r = 0.347, *p* < 0.001; SVR48: r = 0.332, *p* = 0.002).

At baseline, ELF score and TE showed a poor concordance, with Cohen’s kappa coefficient of 0.11 (95% confidence interval −0.11 to 0.35; *p*-value = 0.086) (Table 2). In particular, 102 (98.1%) patients showed, at baseline, advanced liver fibrosis according to TE, whereas 92 (88.5%) subjects had severe liver fibrosis, according to ELF test.

**TABLE 2:** Concordance between ELF and transient elastography at baseline, SVR24, and SVR48.

Baseline				
		ELF		Total
		F1-F2	F3-F4	
Transient elastography	F1-F2	1 (1%)	1 (1%)	2 (1.9%)
	F3-F4	11 (10.6%)	91 (87.5%)	102 (98.1%)
Total		12 (11.5%)	92 (88.5%)	104 (100%)
Cohen’s kappa coefficient: 0.11; 95% confidence interval: −0.11 to 0.35; *p*-value 0.086				
SVR24				
Transient elastography	F1-F2	13 (12.5%)	23 (22.1%)	36 (34.6%)
	F3-F4	15 (14.4%)	53 (51%)	68 (65.4%)
Total		28 (26.4%)	76 (73.1%)	104 (100%)
Cohen’s kappa coefficient: 0.15; 95% confidence interval: −0.05 to 0.34; *p*-value 0.124				
SVR48				
Transient elastography	F1-F2	22 (25.3%)	21 (24.1%)	43 (49.4%)
	F3-F4	9 (10.3%)	35 (40.2%)	44 (50.6%)
Total		31 (35.6%)	56 (64.4%)	87 (100%)
Cohen’s kappa coefficient: 0.34; 95% confidence interval: 0.12 to 0.50; *p*-value 0.003				

At SVR24, the concordance between the two methods remained poor, similar to that at baseline, with Cohen’s kappa coefficient of 0.15 (95% confidence interval −0.05 to 0.34; *p*-value = 0.124) (Table 2). Notably, 68/104 (65.4%) patients were classified as having severe liver fibrosis at TE, and 76/104 (73.1%) showed the same grade of liver fibrosis on the ELF test. Regarding F1-F2 fibrosis, 36 (34.6%) and 28 (26.9%) patients showed mild/moderate fibrosis at TE and ELF, respectively.

At SVR48, the concordance between ELF and TE increased to 0.34 (95% CI: 0.12 to 0.50; *p* = 0.003) (Table 2), with 22 (25.3%) patients showing a mild/moderate fibrosis and 35 (40.2%) a severe fibrosis according to both ELF and TE.

### Time trends of enhanced liver fibrosis and transient elastography from baseline to SVR24 and SVR48

To evaluate time trends of the fibrosis, the analysis was conducted on 96 subjects who performed TE at baseline and at SVR24 and in 119 patients with ELF determination for both times.

**FIGURE 1:**
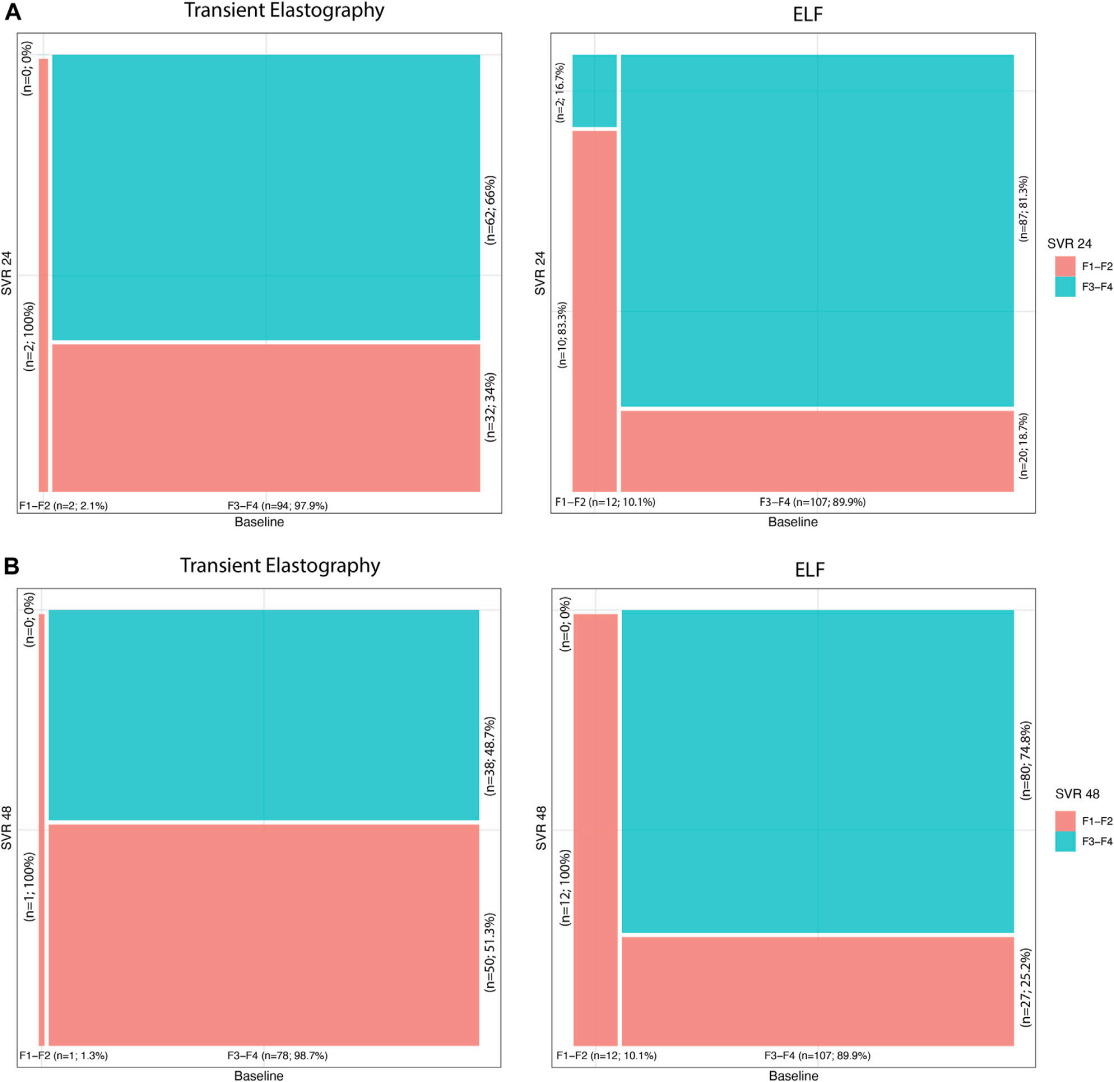
**(A)** Transient elastography and ELF time trends from baseline to SVR24; **(B)** transient elastography and ELF time trends from baseline to SVR48.

Both TE values and ELF test results showed a significant reduction at SVR24 compared to baseline, according to the amelioration of liver fibrosis after DAA therapy (Figure 1A).

At baseline, two (2.1%) patients showed mild/moderate fibrosis and 94 (97.9%) subjects showed advanced fibrosis, according to TE. Instead, at SVR24, 34 (35.4%) patients had F1-F2 values, and 62 (64.6%), F3-F4 fibrosis, with statistically significant variation from baseline (*p*-value <0.001).

Similar amelioration of liver fibrosis was detected also considering the ELF test. Indeed, at baseline, 12 (10.1%) patients showed F1-F2, and 107 (89.9%), F3-F4 fibrosis, while at SVR24, 30 (25.2%) and 89 (74.8%) patients showed mild/moderate and advanced fibrosis, respectively. The decline of ELF was significant (*p*-value <0.001) compared to baseline.

In addition, at SVR48 119 patients were evaluated with ELF scores, and 79 patients, with TE. At baseline, 1 (1.3%) of those patients showed mild/moderate fibrosis, and 78 (98.7%) subjects, advanced fibrosis, according to TE. After 48 weeks of DAA treatment (Figure 1B), 41 (51.9%) patients had F1-F2 values, and 38 (48.1%), F3-F4 fibrosis, with statistically significant variation from baseline (*p*-value <0.001). As for SVR24, the ELF score showed an improvement in the patient’s liver fibrosis. At SVR48, 39 (32.8%) and 80 (67.2%) patients showed mild/moderate and advanced fibrosis, respectively. The decrease in ELF score value was significant (*p*-value <0.001) compared to baseline.

### Enhanced liver fibrosis score and transient elastography in patients with portal hypertension, varices, and ascites

As shown in Table 3, both TE and ELF scores were significantly elevated in HCV patients with PH in comparison to subjects without PH at basal *p* < 0.001 and *p* = 0.010, respectively. Severe fibrosis (F3/F4) was significantly more prevalent in HCV patients with PH based on ELF score (*p* = 0.029), but not on TE (*p* = 0.507). Conversely, there was no significant difference in TE and ELF scores between HCV patients with varices and ascites (data not shown).

**TABLE 3:** ELF score and TE in patients with portal hypertension (PH).

		Portal hypertension		*p*-value
		No (*n* = 60; 50.4%)	Yes (*n* = 59; 49.6%)	
Fibroscan	Score	14.6 [11.9; 20.8] (4.7–33.3)	24 [17.3; 32.4] (10.3–70.6)	<0.001
	F1/F2	2 (3.3)	0 (0)	0.507
	F3/F4	58 (96.7)	44 (100)	
ELF	Score	10.8 [9.9; 11.7] (8.5–14.4)	11.5 [10; 12.6] (8.5–14.4)	0.01
	F1/F2	10 (16.7)	2 (3.4)	0.029
	F3/F4	50 (83.3)	57 (96.6)	

## Discussion

Liver health is a major concern in HCV-infected patients (Monga et al., 2001). Thus, an accurate assessment of liver fibrosis degree is required for clinical decision-making. Liver biopsy is the gold standard (Rockey et al., 2009); however, this is an invasive procedure, with significant variability and a substantial lack of standardization. TE and serological tests represent promising alternative strategies to classify liver fibrosis degree (Day et al., 2019; Ueda et al., 2020). However, TE availability is scarce and requires expensive equipment and trained personnel (17,18). On the other hand, serum biomarkers are inexpensive, safe, and highly reproducible. Studies are required to better evaluate their use in different clinical settings.

In this study, we evaluated the ability of the noninvasive ELF score against the TE to reflect liver fibrosis degree in a cohort of HCV chronic hepatitis before and after treatment with DAAs. The ELF score demonstrated a significant association with stages of liver fibrosis. The score can reliably classify F1-F2 as mild/moderate fibrosis and F3-F4 as advanced fibrosis both at baseline and after therapy.

ELF score directly measures the ECM turnover, and we observed that the ELF score changed in a linear manner with fibrosis degree, indicating its value as a suitable prognostic biomarker to monitor the progression or regression of fibrosis. Accordingly, previous studies showed that ELF test can identify advanced liver fibrosis with good accuracy (Wahl et al., 2012; Lichtinghagen et al., 2013; Agbim and Asrani, 2019). Lichtinghagen et al. (2013) showed that there was a considerable overlap of ELF values, especially in F1-F2, because this test has the highest sensitivity to rule out cirrhosis and not identify the intermediate degree of fibrosis. They sustained that this result suggests that extracellular matrix turnover had a higher influence on fibrosis in moderate stages of liver disease. Therefore, ELF, evaluating markers involved in the synthesis and degradation of extracellular matrix, does not clearly evaluate the intermediate degree of liver fibrosis. Wahl et al. (2012) also showed that both TE and ELF had the highest diagnostic accuracy in predicting advanced fibrosis. Thus, to better discriminate intermediate stages of fibrosis, Agbim and Asrani (2019) suggested the combination of noninvasive serum tests and imaging approaches. Notably, Sherman et al. (2019) showed that patients treated with a blockade agent reduced their ELF scores over time with respect to controls (Sherman et al., 2019).

Several authors reported that ELF score was a predictor of liver disease outcome in subjects with chronic pathologies (Parkes et al., 2010; Day et al., 2019) and of mortality in HIV/HCV-coinfected women (Peters et al., 2016).

The identification of severe hepatic fibrosis is fundamental in HCV-treated patients since cirrhotic patients need to be managed in a manner different from that of non-cirrhotic subjects. Indeed, cirrhotic patients must perform semi-annual ultrasound follow-up for HCC screening, endoscopic surveillance for portal hypertension, and outpatient visits to detect early signs of hepatic decompensation (European, 2018).

It is well known that HCV patients treated with DAAs showed a significant regression of liver fibrosis as a consequence of reduced inflammation linked to the elimination of viral replication (Chekuri et al., 2016; Facciorusso et al., 2018; Rout et al., 2019). In our cohort, both ELF score and TE point out the improvement of liver fibrosis at SVR24 compared to baseline, with a significant time variation at both SVR24 and 48. The percentage of patients who improved ELF scores during follow-up was higher than the percentage of patients who improved liver stiffness. This result suggests that ELF score could detect the DAA-related improvement of liver function before liver stiffness. Our data indicate that ELF score could be useful for the follow-up of these patients. A further larger study population need to be carried out in order to clearly assess the sequential use of ELF and TE.

DAAs are highly effective and well-tolerated and require shorter treatment duration and simpler administration leading to a simplification of HCV treatment, which includes reduced testing for HCV RNA load. In the context of the “simplification era” of DAAs, ELF could play a key role to estimate liver fibrosis stage, avoiding the use of more expansive and time-consuming liver fibrosis monitoring techniques. It is worth noting that ELF score is calculated on instruments used for routine tests, available in several laboratories in many countries. Therefore, it is not necessary to purchase expensive instruments and staff training, and it is sufficient for the availability of ELF score kits to perform the assay.

A likely scenario could be the use of ELF as a screening test in the primary care setting. In the case of ELF negative results, the risk of high-degree fibrosis is low. Conversely, if the ELF test is positive for advanced liver fibrosis, the patient should be taken to the hospital and to undergo further expensive and invasive procedures. ELF score could be useful for the follow-up in patients with high BMI, ascites, severe hepatic inflammation, and hepatic congestion, where TE showed failure rates ranging from 6% to 23% (Horowitz et al., 2017; Agbim and Asrani, 2019).

Finally, ELF score helps avoid unnecessary contacts between the operator and the patients. This scenario fits well with the current SARS-CoV-2 pandemic. In the last 2 years, the diagnosis and treatment of HCV infections have been frequently missed with a high impact in the next years on deaths due to HCV liver diseases and with lengthening of times required to eliminate HCV, as recommended by the WHO (Kondili et al., 2021). Thus, it is essential to start again with the path of eradication of HCV and at the same time, implement all the necessary security measures and avoid unnecessary contacts. The use of this “biological” approach instead of a “physical approach” for the measurement of liver fibrosis in HCV patients can reduce the physical contact between the patients and the medical staff while obtaining an adequate stratification of the fibrosis stage to program a personalized follow-up. Furthermore, the ELF test is a cost-effective, readily available method in low-income countries as well, where HCV infection is more prevalent. Indeed, the requirement of a blood sample and no need for expensive investments in exclusive equipment give a chance to patients living in rural and remote areas in limited-resource countries with better healthcare management in the diagnosis and follow-up of liver fibrosis (Omran et al., 2018).

Our study has some limitations such as the small study population and the low number of patients with low fibrosis stage, but the main one is the lack of the result of the gold standard of histological staging. Nevertheless, others have previously studied ELF score diagnostic and prognostic performance compared to imaging or other serological tests but not to histological findings (French et al., 2016; Peters et al., 2016; Swanson et al., 2016).

## Conclusion

In conclusion, our findings support the use of ELF tests in routine clinical practice for the detection of advanced liver fibrosis in HCV patients before and after DAAs. As a noninvasive test, ELF can avoid unnecessary access to hospitals, allowing the identification of high-risk patients. Notably, this strategy could be used to estimate liver fibrosis before and after DAAs therapies in the context of the COVID-19 pandemic.

## Data Availability

The raw data supporting the conclusions of this article will be made available by the authors, without undue reservation.
