# Supplementary material for: Enhanced liver fibrosis score as a noninvasive biomarker in hepatitis C virus patients after direct-acting antiviral agents
Source: Front Pharmacol. 2022 Aug 17;13:891398. doi: 10.3389/fphar.2022.891398 (PMC9428144; doi:10.3389/fphar.2022.891398)
Supplement: Supplementary file 1 [file Table1.DOCX]

| **Antiviral treatment** | **Patients, n (%)** |
| --- | --- |
| Sofosbuvir+simeprevir ± ribavirin | 14 (11.7) |
| Sofosbuvir/ledipasvir ± ribavirin | 36 (30.3) |
| Sofosbuvir + ribavirin | 20 (16.8) |
| Ombitasvir/paritaprevir/ritonavir ± ribavirin | 44 (36.9) |
| Daclatasvir+sofosbuvir ± ribavirin | 5 (4.3) |

**Supplementary Table 1**. DAA regimens of 119 patients enrolled
